# Transcriptional Events during the Recovery from MRSA Lung Infection: A Mouse Pneumonia Model

**DOI:** 10.1371/journal.pone.0070176

**Published:** 2013-08-01

**Authors:** Jiwang Chen, Gang Feng, Qiang Guo, Juliane B. Wardenburg, Simon Lin, Ichiro Inoshima, Ryan Deaton, Jason X. J. Yuan, Joe G. N. Garcia, Roberto F. Machado, Michael Otto, Richard G. Wunderink

**Affiliations:** 1 Department of Medicine, Northwestern University, Chicago, Illinois, United States of America; 2 Section of Pulmonary, Critical Care Medicine, Allergy and Sleep, University of Illinois at Chicago, Chicago, Illinois, United States of America; 3 Northwestern University Biomedical Informatics Center, Chicago, Illinois, United States of America; 4 Department of Pediatrics, University of Chicago, Chicago, Illinois, United States of America; 5 Biomedical Informatics Research Center, Marshfield Clinic Research Foundation, Marshfield, Wisconsin, United States of America; 6 Department of Pathology, University of Illinois at Chicago, Chicago, Illinois, United States of America; 7 National Institute of Allergy and Infectious Diseases, Bethesda, Maryland, United States of America; Columbia University, United States of America

## Abstract

Community associated methicillin-resistant *Staphylococcus aureus* (CA-MRSA) is an emerging threat to human health throughout the world. Rodent MRSA pneumonia models mainly focus on the early innate immune responses to MRSA lung infection. However, the molecular pattern and mechanisms of recovery from MRSA lung infection are largely unknown. In this study, a sublethal mouse MRSA pneumonia model was employed to investigate late events during the recovery from MRSA lung infection. We compared lung bacterial clearance, bronchoalveolar lavage fluid (BALF) characterization, lung histology, lung cell proliferation, lung vascular permeability and lung gene expression profiling between days 1 and 3 post MRSA lung infection. Compared to day 1 post infection, bacterial colony counts, BALF total cell number and BALF protein concentration significantly decreased at day 3 post infection. Lung cDNA microarray analysis identified 47 significantly up-regulated and 35 down-regulated genes (p<0.01, 1.5 fold change [up and down]). The pattern of gene expression suggests that lung recovery is characterized by enhanced cell division, vascularization, wound healing and adjustment of host adaptive immune responses. Proliferation assay by PCNA staining further confirmed that at day 3 lungs have significantly higher cell proliferation than at day 1. Furthermore, at day 3 lungs displayed significantly lower levels of vascular permeability to albumin, compared to day 1. Collectively, this data helps us elucidate the molecular mechanisms of the recovery after MRSA lung infection.

## Introduction

Community associated methicillin-resistant *Staphylococcus aureus* (CA-MRSA) is an emerging threat to human health throughout the world. Its epidemic spread and high mortality in healthy individuals have raised alarm in the biomedical community. In the USA, CA-MRSA lung infections are almost entirely attributed to a pandemic and highly virulent strain, USA300 [1]–[2]. MRSA pneumonia is characterized by dramatic inflammatory responses in the lungs, resulting in airway neutrophil influx, loss of alveolar architecture, severe lung edema, hemorrhage and intrapulmonary bacterial proliferation [3]–[5].

A combination of pathogen virulence factors and host factors determine the development of diseases in healthy subjects exposed to MRSA. Some studies have identified host and bacterial factors facilitating progression of MRSA pneumonia. Montgomery *et al*. (2009) employed a rat necrotizing MRSA-mediated pneumonia model to study the transcription of 84 genes mediating the early inflammatory response in the lung [5]. Ventura *et al*. (2008) utilized a mouse model of MRSA pneumonia to define the early events in the innate immune response and assess the changes in the airway proteome during the first 6 hours (h) of pneumonia [6]. In both rats [5] and mice [6], the expression of cytokines and chemokines was greatest 6 h after inoculation of MRSA and decreased thereafter. In addition to inflammatory proteins, antimicrobial peptides, opsonins, and coagulation proteins also dominate the early response to MRSA pneumonia. In a sublethal dose of MRSA-mediated mouse pneumonia model, the mice were able to clear infection within 24 to 36 h, and the infected lungs started to recover [7]. Later events in the recovery phase after MRSA lung infection are largely unknown.

In this study, an established sublethal mouse model of MRSA pneumonia and genome – wide transcriptional profiling were employed to compare lung gene expression patterns between days 1 (24 h) and 3 (72 h) post infection. In addition, we studied changes of lung bacterial clearance, bronchoalveolar lavage fluid (BALF) protein concentration, BALF total cell number, lung histological features, lung permeability and cell proliferation in this process.

## Materials and Methods

### Bacterial Preparation for Inoculation

USA300 MRSA wild-type strain (LAC) was used in this study. To prepare an inoculum for animals, a frozen stock of MRSA LAC strain was incubated onto a tryptic soy agar plate at 37°C overnight. Three ml tryptic soy broth (TSB) was inoculated from a single colony and incubated overnight in a shaker set at 250 rpm and 37°C. One ml of overnight culture was then grown in 100 ml TSB solution until an optical density (OD) at 660 nm of approximately 0.5 was reached. Fifty ml of bacterial culture was then centrifuged at 6000 g for 15 min at 4°C, washed in sterilized phosphate-buffered solution (PBS) and resuspended in 1.5 ml PBS solution, resulting in an estimated concentration of 1×10^8^ CFU per 30 µl. The solution was immediately used for animal inoculation. All inocula were quantified by plating serial dilutions in PBS on tryptic soy agar and counting colonies after overnight incubation at 37°C.

### Animals and Procedures

All animal experiments and procedures were approved by the Institutional Animal Care and Use Committee at Northwestern University. The lung infection procedure described previously [8]–[9] was followed. Briefly, C57Bl6 male mice (7 weeks old, Charles River) were anesthetized before inoculation of 30 µl of MRSA suspension to the left nare. Animals were held upright for one minute post-inoculation and then placed into a cage in supine position for recovery. In the control group, mice were inoculated with 30 µl of sterilized PBS solution. All animals were provided with food and water *ad libitum* and continually observed for the time courses indicated in the figures. A small number of animal that succumbed within the first 6 hours post infection (<1%) were excluded in the subsequent data analysis.

### Bronchoalveolar Lavage (BAL) Characterization

BAL was performed by instilling one ml of cold Hank’s Salt solution (HBSS, Invitrogen, Grand Island, NY) via tracheal cannula, as previously described [10]. Total cells were counted by TC10™ Automated Cell Counter (BioRad). Differential counts of BAL cells were performed by cytocentrifugation (CytoSpin 3; Shandon Instruments, Pittsburgh, PA) and staining with Diff-Quick (Dade, Behring, Dudinger, Switzerland). BAL protein concentration was measured using a Pierce^®^ BCA protein assay kit (Thermo Scientific Inc, Rockford, IL).

### Assessment of Lung Permeability

The procedure for lung permeability assay described previously [11] was followed. Briefly, one day and three days after intratracheal inoculation of MRSA or PBS, mice were anesthetized and a 20 - gauge angiocath was sutured into the trachea. The mice were mechanically ventilated (Minvent; Harvard Apparatus) with a respiratory rate of 100 and a tidal volume of 0.2 ml. The left external jugular vein was identified into which 0.15 ml of a 16 mg/ml solution of FITC-labeled albumin (Sigma-Aldrich) was performed. Relative lung permeability was estimated from the fluorescence in the BAL fluid measured by using a microplate reader (excitation = 488 nm, emmision = 530 nm).

### Histology Preparation

A 20-gauge angiocath was sutured into the mouse trachea. The lungs were inflated with 0.8 ml 4% paraformaldehyde (PFA) and fixed in a 15 ml tube with the same concentration of PFA solution overnight at 4°C. The fixed lungs were further paraffinized and 5-µm sections were stained with hematoxylin/eosin.

### Microarray Data and Functional Analysis

RNA expression analysis was performed using the Illumina Mouse Ref-8 BeadChip (Illumina, San Diego, CA), which provides coverage of approximately 25,700 genes and expressed sequence tags. Four independent mouse lung tissue samples at days 1 and 3 post MRSA - infected and PBS control groups from four independent experiments were used for RNA isolation with a RNeasy plus mini kit (Qiagen, Valencia, CA). RNA quality was checked using an Agilent Bioanalyzer (Santa Clara, CA) and further labeled using a commercial kit (TargetAmp 1 - Round Aminoallyl-aRNA Kit; Epicentre, Madison, WI, USA). Labeled RNA was then hybridized to the Illumina MouseRef - 8 BeadChip. Raw signal intensities of each probe were obtained using data analysis software (Beadstudio; Illumina) and imported to the *lumi* package of Bioconductor for data transformation and normalization [12]–[14]. Absent/present call detection was performed using a p value of 0.01 as threshold. 15,490 out of 25,697 probes were considered valid signals.

Differentially expressed genes in the lungs between MRSA – infected and PBS control groups at day 1 and day 3 separately were identified using an Analysis of Variance (ANOVA) model with empirical Bayesian variance estimation [15]. The problem of multiple comparisons was corrected using the false discovery rate (FDR). To reduce false positives, genes above-background in at least one experiment were used for subsequent analysis. Stringent criteria (fold change ≥1.5 up or down, *p*<0.01, FDR <0.05) were used to filter differentially expressed genes. Two-dimensional hierarchical clustering was applied to these filtered probes to generate a global overview of the gene expression map (heat map).

Functional analyses of the significantly differentially expressed genes were performed by GeneAnswers package of Biocoductor based on hypergeometric test on Gene Ontology [16]–[18]. Microarray data was deposited in the Gene Expression Omnibus database with accession number GSE #36587 (GEO database, http://www.ncbi.nlm.nih.gov/geo/).

### Reverse Transcription and Real-time PCR Validation

Real-time PCR data were from six experiments including the four experiments used for microarray data analysis. Two µg of purified RNA was reverse transcribed to single-strand cDNA using Taqman RNA reverse transcription kit (cat. # N8080234, Applied Biosystems Inc [ABI]). Real-time PCR was performed on an ABI 7900HT machine. According to lung cDNA microarray data, four up-regulated (cdc20, Apoe, Thy1, Anln) and four down-regulated genes (Orm1, Serpina3m, JunB, Egr1) between Day 1 and Day 3 post infection were selected for real-time PCR validation. Specific real-time PCR assays for the eight genes are summarized in Table 1. The mRNA expression levels were normalized to the expression of a housekeeping gene, hexose-6-phosphate dehydrogenase (G_6_PDH). Fold change was analyzed using the 2^−ΔΔCt^ method, where ΔΔC_t_ = (C_t, target_ – C_t, G6PDH_)_Day 3_ - (C_t, target_ – C_t, G6PDH_)_Day 1_.

**Table 1:** Taqman gene expression assay ID numbers for the genes which were significantly regulated during the recovery after MRSA lung infection.

gene ID	Assay ID
anln	Mm01170327_m1
apoe	Mm01307192_m1
Cdc20	Mm00650983_g1
Egr1	Mm00656724_m1
JunB	Mm01251660_s1
Orm1	Mm00435456_g1
Serpin3f	Mm02763616_s1
Thy1	Mm01174153_m1
H_6_PDH	Mm00557617_m1

These gene expression ID number can be found in https://products.appliedbiosystems.com **.**

### Lung Cell Proliferation Assay

To compare lung cell proliferation between days 1 and 3 post MRSA lung infection, expression of PCNA (proliferating cell nuclear antigen) was evaluated. Lung tissue sections were deparaffinized in xylene, and then incubated with a graded series of alcohol, rehydrated in PBS (pH 7.4). Antigen retrieval was performed in 1 mM Tris (pH 9.0) with 0.2% tween 20 at 95°C for 20 min. Slides were then washed in PBS, and incubated in a blocking solution (10% normal goat serum with 0.1% TX-100 in PBS). The slides were further incubated overnight at 4°C with a rabbit anti-PCNA antibody (1∶100, Santa Cruz Biotechnology, Inc). The slides were washed in PBS with 0.1% TX-100 three times for 10 min, and then incubated with Alexa 488-conjugated donkey anti-rabbit antibodies for one hour at room temperature. After washing, the slides were fixed with a DAPI-mounting solution (Invitrogen Inc). PCNA-DAPI staining images were examined in a Zess Immunofluorescence microscope. PerkinElmer InForm version 1.3.0 software was used to calculate the percentage of PCNA positive cells in each image.

### Statistical Analysis

Two-tailed Student *T-test* was used for comparisons between two groups. P<0.05 was considered statistically significant. The results are shown as the means with error bars depicting ± standard error of mean (SEM) for at least three independent experiments. All statistical analysis was performed using GraphPad Prism 5.1 (GraphPad Software, La Jolla, CA).

## Results

### MRSA Clearance in Lungs at Days 1 and 3 Post Infection

**Figure 1:**
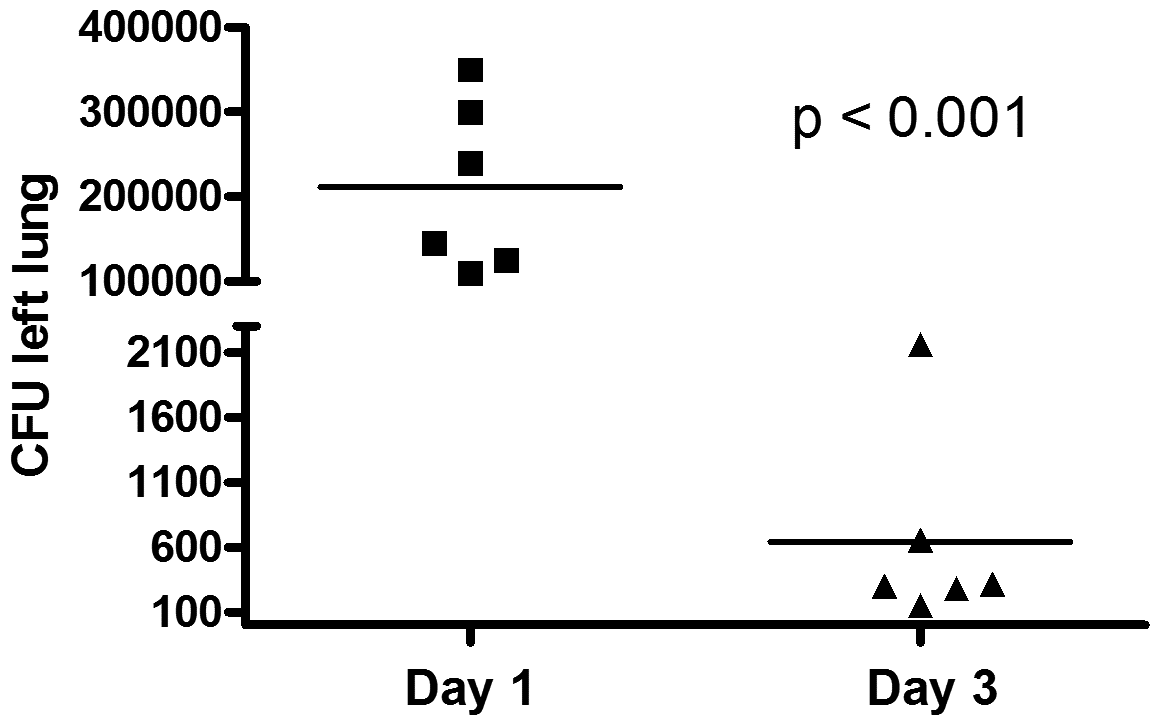
Bacterial burden in the lungs significantly decreased at day 3 compared to day 1 post MRSA infection (p<0.001). Animals (n = 6 per group) were inoculated with 1.0×10^8^ CFU of MRSA (LAC strain), and the bacterial CFU in left lungs (24 and 72 h) were enumerated at days 1 and 3 post infection. Data were analyzed from three independent experiments to determine significance using Student’s t-test.

Mice (n = 6 per group) were inoculated with 1.0×10^8^ CFU of MRSA (LAC strain). The bacterial burden in the lungs (Figure 1) significantly decreased at day 3 compared to day 1 post MRSA lung infection (day 1: 211700±40840 CFU; day 3: 643±311 CFU, p<0.001).

### Lung Histopathologic Features at Days 1 and 3 Post Infection

**Figure 2:**
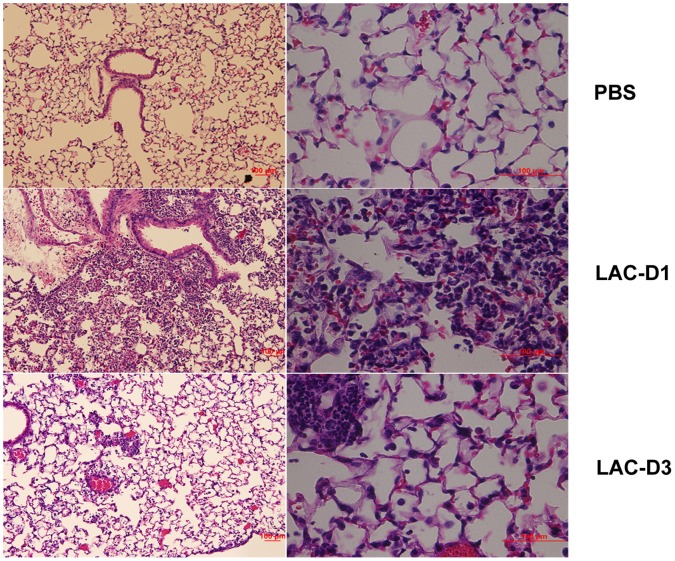
Lung histopathology at days 1 and 3 post MRSA lung infection. A. PBS saline group; B. Day 1 post MRSA lung infection group; C. Day 3 post MRSA lung infection group. Magnification time: × 20.

Hematoxylin/eosin (HE) staining of lung tissues is shown in Figure 2. Compared to the PBS control group (Figure 2A), more inflammation with pulmonary edema, multifocal bacterial aggregates, and lung structure destruction was observed in the lungs at day 1 post MRSA lung infection (Figure 2B). At day 3 post MRSA lung infection, no multifocal bacterial aggregates were found, but inflammatory infiltrates were still seen in the lung alveolar space (Figure 2C).

### Bronchoalveolar Lavage Fluid (BALF) Protein Concentration, Total Cell Number and Cell Differential Staining

**Figure 3:**
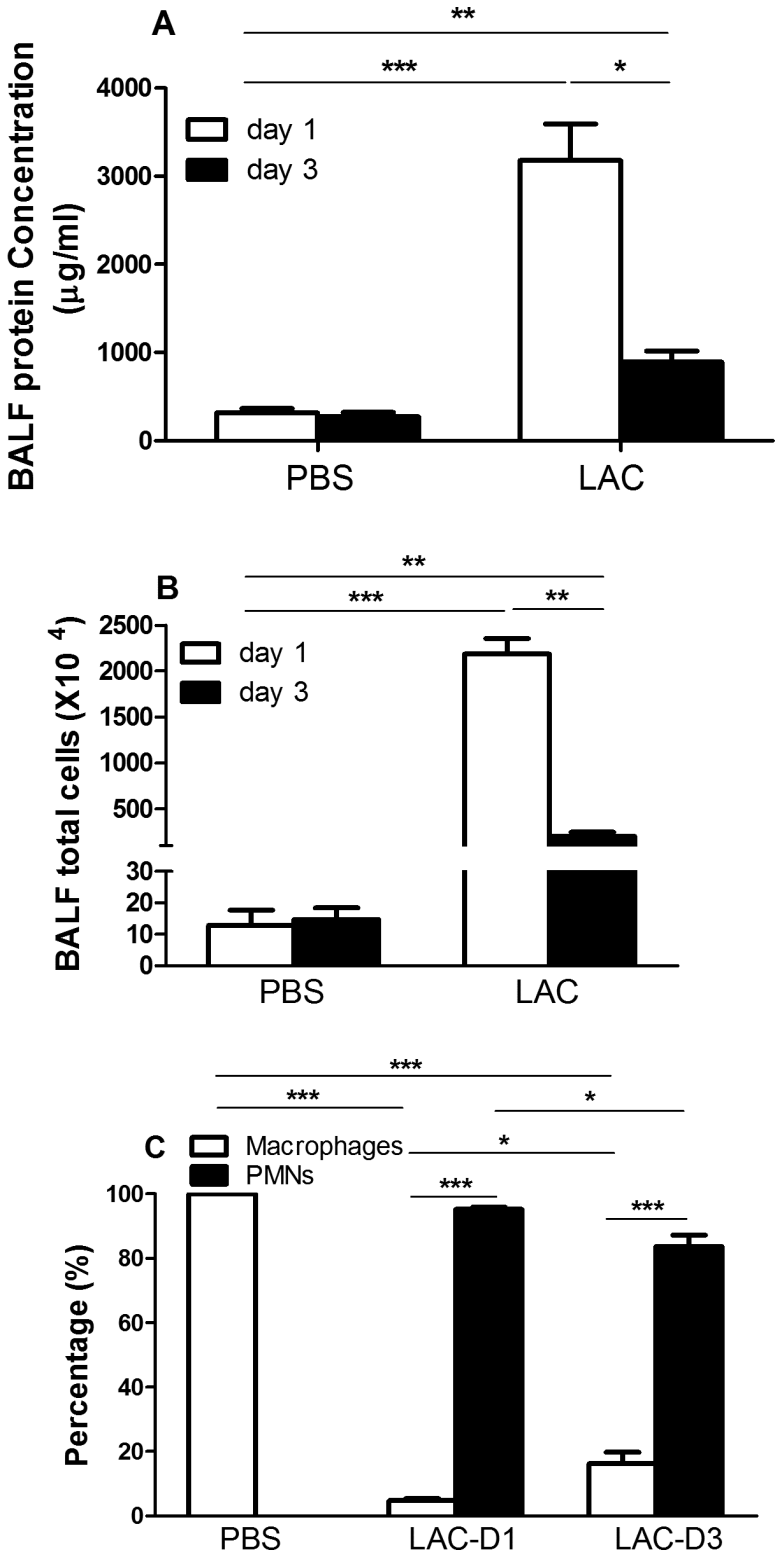
Bronochioalveolar lavage fluid (BALF) protein concentration (A), total cell number (B) and differential staining counts (C) in the lungs at days 1 and 3 post MRSA lung infection or PBS delivery. PMN: polymorphonuclear neutrophils. *, p<0.05; **, p<0.01; ***, p<0.001.

In the MRSA infected group, BALF protein concentration and total cell number significantly decreased at day 3 compared to day 1 (Figure 3A, 3B). In the PBS control group, neither significantly changed. Macrophages dominated in the PBS group (Figure 3C), while polymorphonuclear neutrophils (PMNs) dominated in MRSA-infected group (p<0.001). During the recovery phase, the percentage of PMNs in BALF significantly decreased at day 3 in the infected group, compared to day 1 (p<0.05). The percentage of macrophages significantly decreased at day 1, but significantly increased at day 3 during the recovery, compared to day 1 (p<0.05).

### Lung Vascular Permeability Recovered at Day 3 Post MRSA Lung Infection

**Figure 4:**
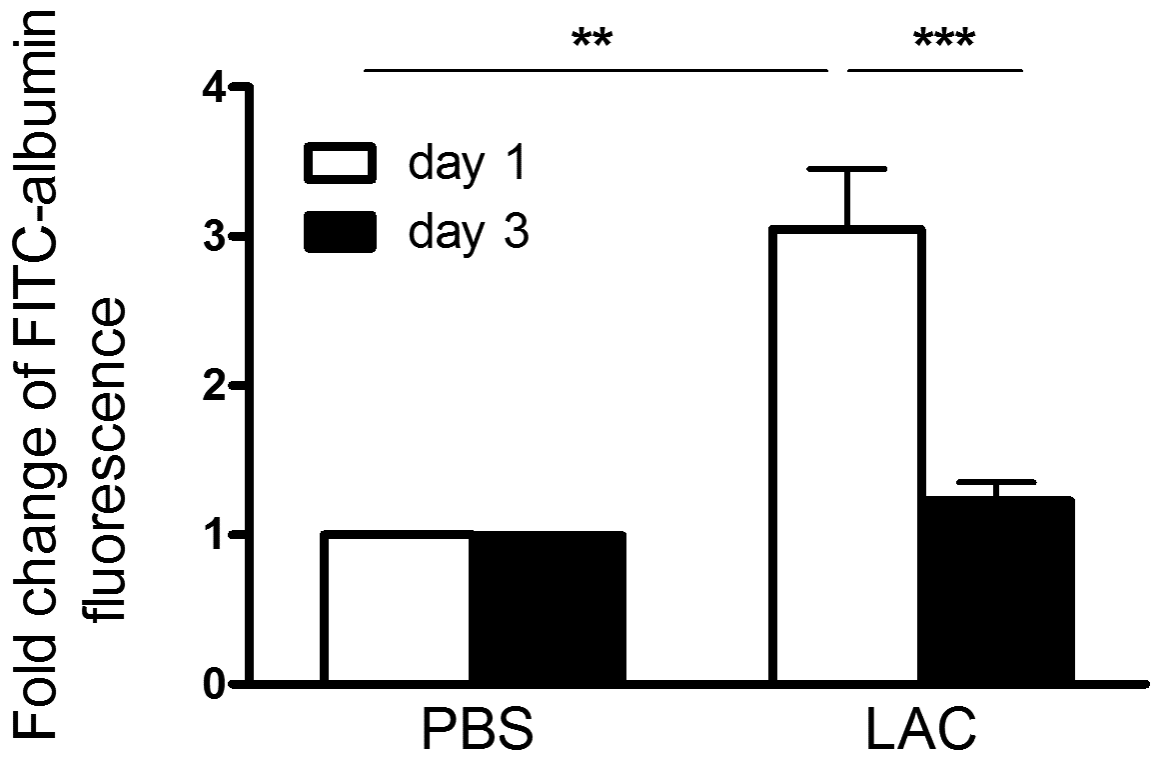
Fold change for lung permeability to FITC-labeled albumin at days 1 and 3 post MRSA lung infection. Data was from three independent experiments. *, p<0.05; **, p<0.01; ***, p<0.001.

Compared to the PBS group, the day-1 post MRSA lung infection group displayed significantly increased levels of lung permeability to albumin. However, at day 3 post MRSA lung infection, lung vascular permeability dropped to the levels of the PBS control (p<0.001, compared to day 1, Figure 4).

### Global Gene Expression Change during the Recovery Post MRSA Lung Infection

**Figure 5:**
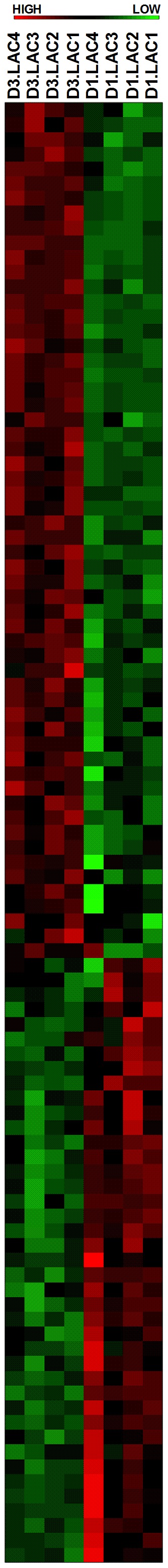
Heat map analysis reveals a global view of genes up- and down-regulated in lungs between Day 1 and Day 3 post MRSA lung infection. The 82 differentially expressed genes were used to construct this heatmap.

To explore changes in global gene expression change during the recovery phase after MRSA lung infection, cDNA microarray analysis was conducted with lung samples obtained from the mice after days 1 and 3 post infection. Overall, we observed marked differences in gene expression patterns between day 1 and day 3 groups (Figure 5). It also indicated, in a global view, highly consistent results between the four replicates in each group (Figure 5). Of 82 differentially expressed genes (Table S1), 47 (57%) were up-regulated and 35 (43%) were down-regulated. Table 2 and Table 3 list the top 30 transcripts up-regulated and down-regulated during the recovery after MRSA lung infection, respectively.

**Table 2:** The list of top 30 transcripts up-regulated during the recovery from MRSA lung infection.

Symbol	Description	Fold Change	EntrezID
**Ms4a7**	membrane-spanning 4-domains, subfamily A, member 7	6.5	109225
**Prc1**	protein regulator of cytokinesis 1	5.5	233406
**Cenpa**	centromere protein A	4.7	12615
**Top2a**	topoisomerase (DNA) II alpha	4	21973
**Cdc20**	cell division cycle 20 homolog (S. cerevisiae)	4	107995
**Hist1h2ad**	histone cluster 1, H2ad	3.9	319165
**Cdk1**	cyclin-dependent kinase 1	3.9	12534
**C1qc**	complement component 1, q subcomponent, C chain	3.9	12262
**Apoe**	apolipoprotein E	3.8	11816
**Cd3g**	CD3 antigen, gamma polypeptide	3.8	12502
**Pbk**	PDZ binding kinase	3.8	52033
**Hist1h2ah**	histone cluster 1, H2ah	3.8	319168
**Mfap4**	microfibrillar-associated protein 4	3.7	76293
**Lyz1**	lysozyme 1	3.7	17110
**Nusap1**	nucleolar and spindle associated protein 1	3.6	108907
**Thy1**	thymus cell antigen 1, theta	3.5	21838
**Cx3cr1**	chemokine (C-X3-C) receptor 1	3.5	13051
**C1qb**	complement component 1, q subcomponent, beta polypeptide	3.4	12260
**Gpnmb**	glycoprotein (transmembrane) nmb	3.4	93695
**Slamf9**	SLAM family member 9	3.3	98365
**Trem2**	triggering receptor expressed on myeloid cells 2	3.2	83433
**2810417H13Rik**	RIKEN cDNA 2810417H13 gene	3.2	68026
**Birc5**	baculoviral IAP repeat-containing 5	3.1	11799
**Ctsk**	cathepsin K	2.9	13038
**Ctsa**	cathepsin A	2.9	19025
**D0H4S114**	DNA segment, human D4S114	2.9	27528
**Ccno**	cyclin O	2.9	218630
**Ly86**	lymphocyte antigen 86	2.9	17084
**Kif23**	kinesin family member 23	2.8	71819
**Anln**	anillin, actin binding protein	2.8	68743

**Table 3:** The list of top 30 transcripts down-regulated during the recovery from MRSA lung infection.

Symbol	Description	Fold Change	EntrezID
**Ccl4**	chemokine (C-C motif) ligand 4	−5.1	20303
**Orm1**	orosomucoid 1	−4.7	18405
**Timp1**	tissue inhibitor of metalloproteinase 1	−4.6	21857
**Angptl4**	angiopoietin-like 4	−4.4	57875
**Gdf15**	growth differentiation factor 15	−4	23886
**Ubd**	ubiquitin D	−3.9	24108
**Orm2**	orosomucoid 2	−3.8	18406
**Serpina3m**	serine (or cysteine) peptidase inhibitor, clade A, member 3M	−3.7	20717
**Il1rn**	interleukin 1 receptor antagonist	−3.6	16181
**S100a9**	S100 calcium binding protein A9 (calgranulin B)	−3.4	20202
**Cxcl1**	chemokine (C-X-C motif) ligand 1	−3.2	14825
**Socs3**	suppressor of cytokine signaling 3	−3.1	12702
**Upp1**	uridine phosphorylase 1	−3	22271
**Il1r2**	interleukin 1 receptor, type II	−3	16178
**Junb**	Jun-B oncogene	−3	16477
**Mt2**	metallothionein 2	−2.8	17750
**Il4i1**	interleukin 4 induced 1	−2.8	14204
**Tinagl1**	tubulointerstitial nephritis antigen-like 1	−2.7	94242
**Bach1**	BTB and CNC homology 1	−2.7	12013
**Ifitm6**	interferon induced transmembrane protein 6	−2.7	213002
**Ier3**	immediate early response 3	−2.7	15937
**Tap2**	transporter 2, ATP-binding cassette, sub-family B (MDR/TAP)	−2.7	21355
**Wars**	tryptophanyl-tRNA synthetase	−2.6	22375
**Csrnp1**	cysteine-serine-rich nuclear protein 1	−2.6	215418
**Gadd45b**	growth arrest and DNA-damage-inducible 45 beta	−2.5	17873
**Tnfaip2**	tumor necrosis factor, alpha-induced protein 2	−2.5	21928
**Atf5**	activating transcription factor 5	−2.5	107503
**Egr1**	early growth response 1	−2.4	13653
**Gadd45g**	growth arrest and DNA-damage-inducible 45 gamma	−2.3	23882

**Figure 6:**
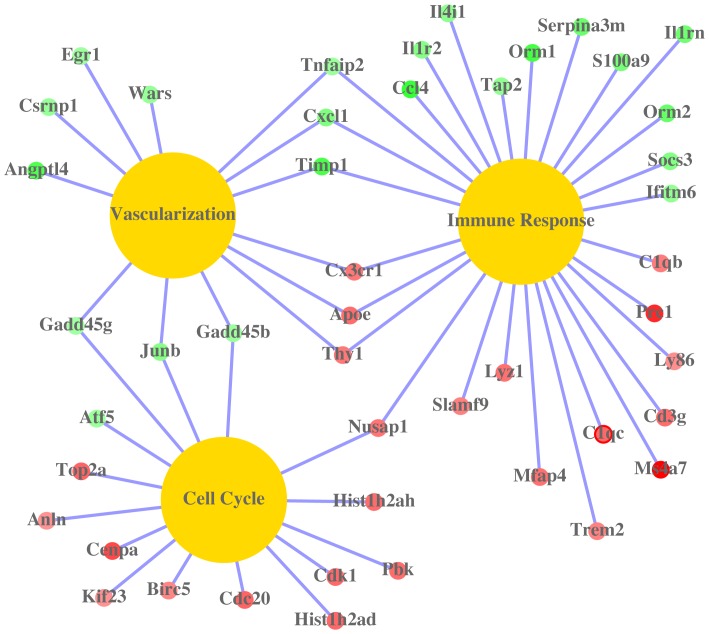
The relationship of the top up - and down - regulated genes and gene ontology categories (immune response, vasculariation and cell cycle) during the recovery post MRSA lung infection. Yellow nodes represent gene ontology categories, red nodes are up-regulated genes, while green nodes stand for down-regulated genes. The saturations of gene nodes are proportional to the fold changes of these genes during the recovery post MRSA lung infection.

Assignment of the significantly altered genes into the biological-process GO category reveals the following three essential lung and cellular functional regulations which contribute to recovery post MRSA lung infection, which are highlighted in Figure 6:

1. Readjusting host immune responses. Chemokine (C-C motif) ligand 4 [ccl4, also called macrophage inflammatory protein-1β (MIP-1β)] was the most significantly down-regulated gene during the recovery from infection (Table 3). Ccl4 is a chemoattractant for natural killer cells including neutrophils [19]. Another neutrophil chemoattractant, Cxcl1 (chemokine [C-X-C motif] ligand 1) [20], was also down-regulated. Down-regulation of these proteins may prevent further recruitment of neutrophils, which produce ROS and inflammatory cytokine and chemokines. Accordingly, some ROS or cytokine/chemokine induced proteins were also down-regulated. These proteins include type II interleukine receptor (IL1R2), TIMP1 (a tissue inhibitor of metalloproteinases), wars (a cytoplasmic form of tryptophanyl-tRNAsynthetase), Gadd45b, Gadd45g and SOCS3 (suppressor of cytokine signaling 3) [21]–[26]. Acute-phase proteins, including Orm1 (alpha-1-acid glycoprotein 1), Orm2, Serpina3m (serine or cysteine peptidase inhibitor), ler3 (immediate early response 3) and Egr1 (early growth response 1), were also down-regulated during the recovery phase. These acute phase proteins have previously been demonstrated to be elevated in response to MRSA lung infection [27]–[30]. Trem2 (triggering receptor expressed by myeloid cells 2) and complement components C1qb and C1qc were up-regulated during the recovery. Trem2 was shown to promote phagocytosis and retard inflammation [31]. Complement plays an essential role in pneumonia. Patients with fulminant disease and signs of septic shock showed pronounced hypocomplementemia [32]. C1q was shown to elicit diverse array of cellular responses including platelet activation, localization of the immune complex by endothelial cells, enhancement of phagocytosis in monocytes, chemotaxis of eosinophils and inhibition of IL-1 synthesis by B lymphocytes [33]–[35]. Up-regulation of C1q complement during the recovery stage may contribute to intravascular coagulation and thrombotic complications.
2. Enhancing cell division and proliferation. Our microarray data show that cdc20, cenpa, top2a, Hist1h2ad, Hist1h2ah, cdk1, Kif 23, pbk, anln and prc1 were significantly up-regulated. These proteins are involved in cell division [36]–[45]. For example, cdc20 (cell-division cycle protein 20) is an essential regulator of cell division by activating the anaphase promoting complex, which initiates chromatid separation and entrance into the anaphase [45]. In addition, some proteins regulating cell proliferation and anti-apoptosis were also up-regulated. These proteins include JunB, Birc5 (baculoviral IAP repeat-containing 5), Apoe (apoliprotein E) and CX3CR1 (CX3 chemokine receptor 1) [46]–[49].
3. Promoting blood vessel and vasculature development. All proteins promoting cell division, as described above, may also contribute to blood vessel and vasculature development via repairing damaged lung endothelium, enhancing cell-cell junctions or promoting smooth muscle cell proliferation. In addition, our microarray data show that thy1 was up-regulated, and Angptl4, Cxcl1, SOCS3, wars, Tnfaip2 were down-regulated. These genes are associated with blood vessel and vasculature development [50]–[56]. For example, Angptl4 (angioproetin-related protein 4) was shown to disrupt endothelial cell-cell junctions by directly interacting with integrin, VE-cadherin and claudin-5 in a sequential manner to facilitate metastasis [52]. Down-regulation of Angptl4 during the recovery after MRSA lung infection may restore lung endothelial barrier functions. Our study showed that lung vascular permeability to albumin was significantly increased at day 1 post MRSA lung infection (Figure 4), which indicates lung vascular endothelium was dampened by MRSA lung infection. However, lung vascular permeability to albumin at day 3 post MRSA lung infection recovered to the level observed in the PBS control group, which suggests that lung vascular endothelium was repaired during the recovery phase.

### Validation of Gene Expression Changes by Real-time PCR

**Figure 7:**
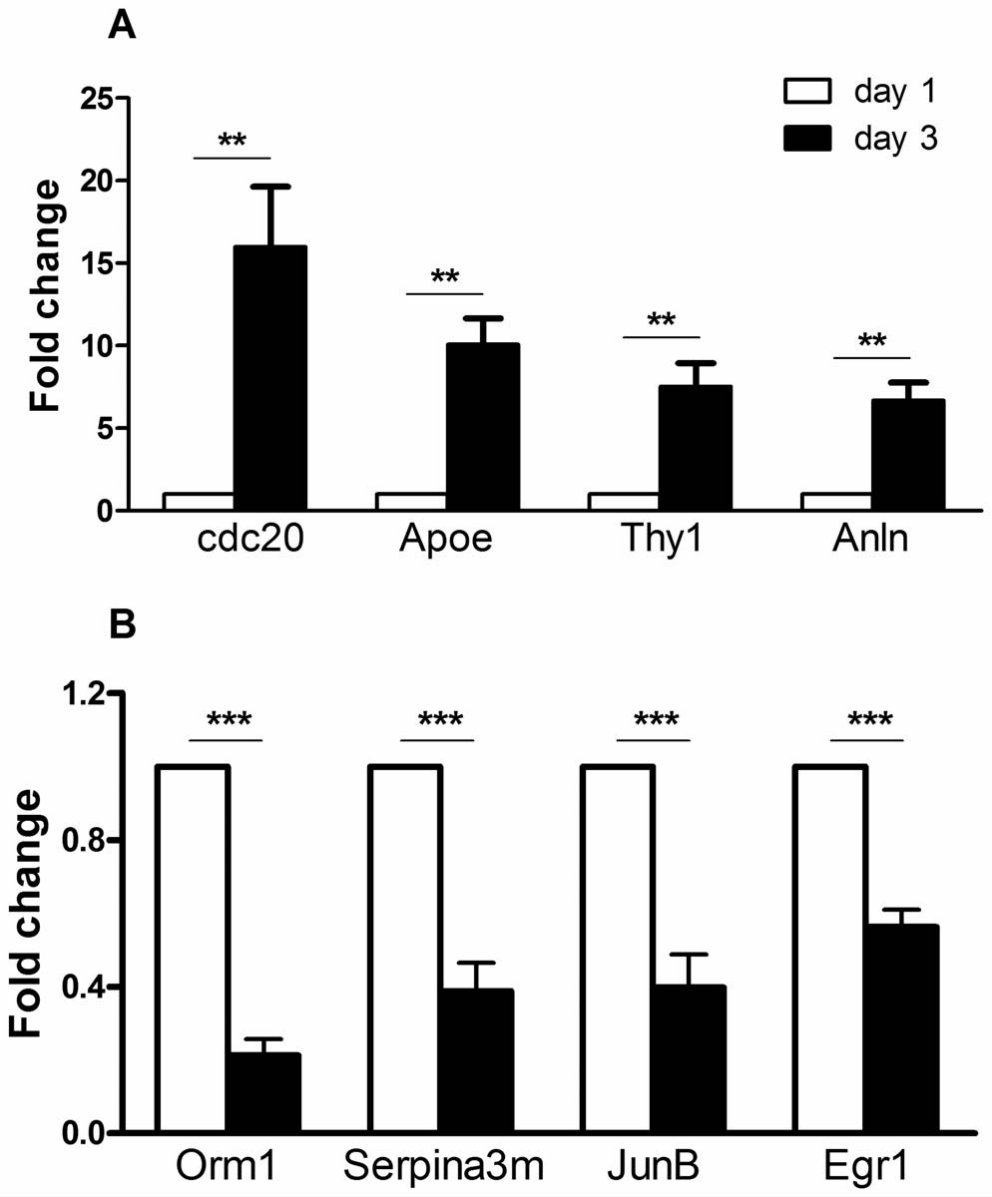
Real-time PCR validation of four up-regulated (A) and four down-regulated (B) genes revealed by microarray hybridization. The *y* axis labeled “fold Change” is defined in the materials and methods section. Blank bars represent Day 1 and black bars represent Day 3. Results are represented as mean ± standard error from six independent experiments including the four experiments for microarray data analysis. Statistical analysis was performed using analysis of variance (ANOVA). ** p<0.01, ***p<0.001.

According to lung cDNA microarray data, eight genes, involved in cell division, vasculature development or host immune response, were selected for verification with real-time PCR using the Taqman gene expression assays (Table 1). The fold changes from real-time PCR data (Figure 7A and 7B) were consistent with cDNA microarray hybridization. The functions of these genes are listed in Table 4.

**Table 4:** The known functions of eight verified genes.

Gene Symbols	Entrez IDs	Gene Names	Functions
Apoe	11816	apolipoprotein E	response to Wounding, blood vessel morphogenesis blood vessel, vasculature and organ development
Anln	68743	anillin, actin binding protein	involved in mitotic cell cycle, mitosis, nuclear division and organelle fission
cdc20	107995	cell-division cycle protein 20	regulation of mitotic cell cycle, nuclear division
			and organelle fission
Thy1	21838	thymus cell antigen 1, theta	blood vessel and vasculature development
			involved in cell-cell and cell-matrix interactions, with implication in apoptosis, inflammation and fibrosis.
Orm1	18405	orosomucoid 1	acute inflammation response, response to wounding; involved in immunosuppression.
Serpina3m	20717	serine peptidase inhibitor, clade A,	protects lower respiratory tract from damage caused by proteolytic enzymes; an acute phase protein induced during inflammation.
		member 3m	
Jun B	16477	Jun B oncogene	regulation of cell cycle and blood vessel morphogenesis, blood vessel, vasculature and organ development.
Egr1	13653	Early growth response 1	a transcriptional regulator; the products of its activated genes are required for differentiation and mitogenesis.

### Lung Cell Proliferation Significantly Increased at Day 3 Post MRSA Lung Infection

**Figure 8:**
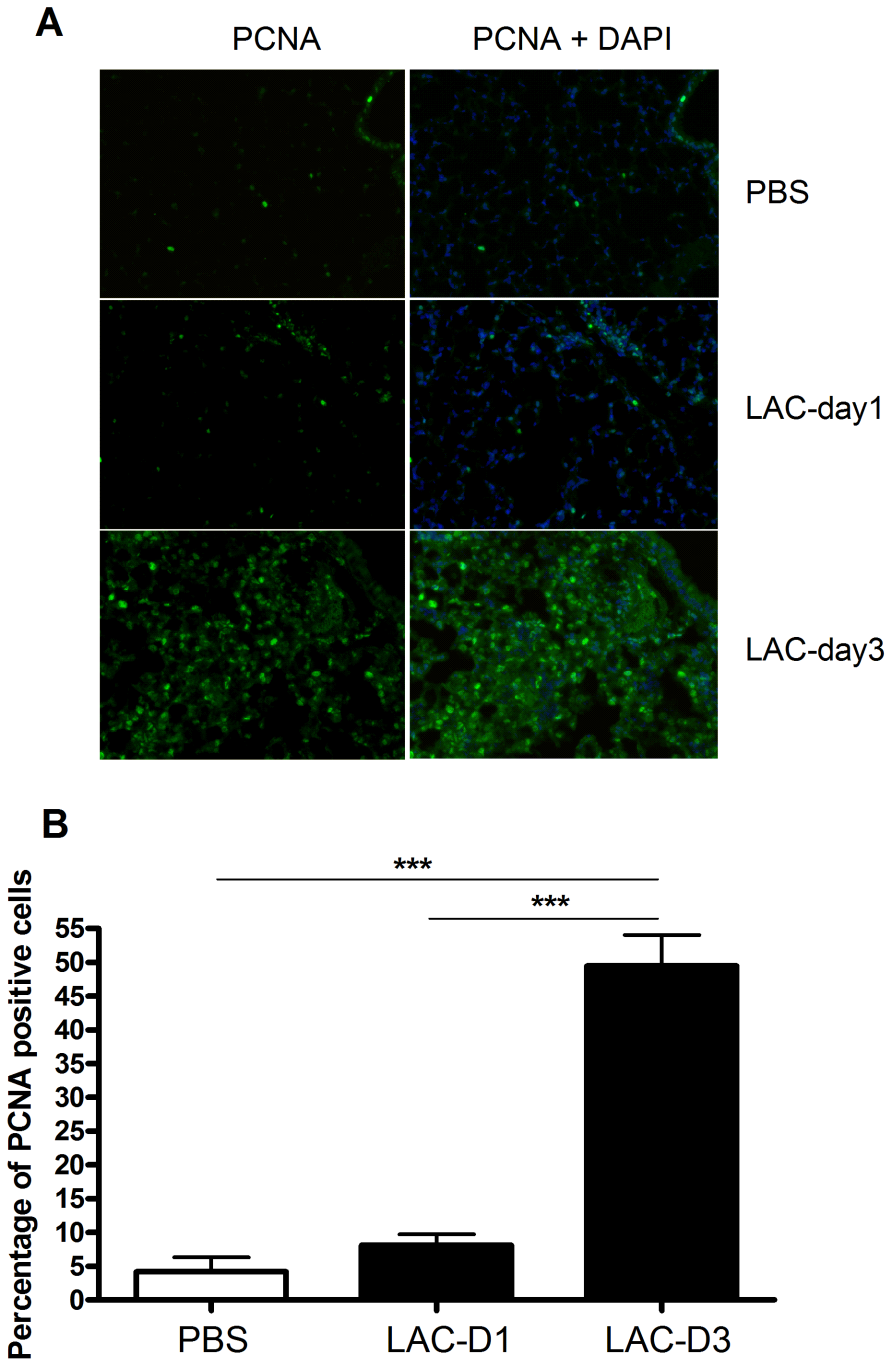
Immunohistochemistry for lung cell proliferation assay in controls (PBS), Day 1 and Day 3 post MRSA lung infection (LAC-D1, LAC-D3). A) Representative PCNA and PCNA-DAPI immunostained mouse lung images at Day 1 and Day 3 post MRSA infections: Green dots are PCNA-positive cells; DAPI-stained blue dots indicate total cell number in one screen. Magnification time: × 20; B) Quantification of PCNA positive cell percentage in the three groups using PerkinElmer InForm version 1.3.0. Data were from three independent experiments, with six images taken from each experiment. ***p<0.001, compared to PBS group.

PCNA immune-staining data (Figure 8) showed that significantly increased cell proliferation occurs in the lungs at day 3 post MRSA lung infection, compared to day 1. At day 1, we also observed increased cell proliferation, as compared to the PBS group, although this was not statistically significant (p = 0.18).

## Discussion

In this study, the transcriptional events in the mouse lung during recovery from MRSA lung infection were investigated using a cDNA microarray. We identified 82 differentially expressed genes; changes in eight of the genes were verified by real-time PCR. Differentially expressed genes were involved in cell division, cell proliferation, blood vessel, blood vasculature and organ development, regulation of host immune response and responses to wounding. In accordance with the transcriptional profiling data, functional studies demonstrated that with prolonged infection, cell proliferation increases and lung vascular permeability to albumin decreases. The data obtained in this study may serve as a starting point for further investigation of the events occurring during the recovery of the lung after MRSA infection. It is possible that modulating expression levels of these differentially regulated genes defined in this study may be able to alter the course of lung infection during MRSA pneumonia.

Our data show that over the course of infection, the BALF total cell number, BALF protein concentration, the percentage of polymorphonuclear neutrophils (PMN) significantly decreased while the percentage of alveolar macrophages significantly increased. It has been reported [57] that the number of alveolar macrophages is well correlated with bacterial burden during lung infection with *Pneumocystis carinii.* A rapid decrease in alveolar macrophage number occurred very early in a rodent model of *Pneumocystis carinii* pneumonia, and the mice had a profound increase of alveolar macrophages during the recovery stage [57]. Our data is consistent with this finding. The increase of percentage of alveolar macrophages at Day 3 post MRSA infection indicates that recovery from pneumonia occurred at that time. In pneumonia, alveolar macrophages play a protective anti-inflammatory role. An in vivo mouse model of klebsiella pneumonia demonstrated that elimination of alveolar macrophages dampens bacterial clearance and survival, although it increases neutrophil recruitment [58].

Transcriptional events in the lung during the recovery in some other disease states have been reported. For example, Chen *et al* (2007) employed a rat model of hyperoxia – mediated lung injury and showed that a series of genes including bmp-4, retnla, meox2, fdps, rgc-32, Dlk1 were up-regulated [59]. These genes are involved in cell proliferation and differentiation, possibly through the modulation and integration of MAPK, TGF-β and other signaling pathways. However, these genes have not been found up-regulated in our pneumonia model, which may be due to different animal models and different samples used. Chen *et al* (2007) studied gene expression patterns in isolated type II alveolar epithelial cells during hyperoxia – mediated lung recovery. Another group employed lung microarray analysis in a rat model of stainless-steel welding fume exposure to examine the transcriptional events during the recovery [60]. These authors demonstrated that some host defense components including Trem2 and some immunoglobulin family members such as IgG-2a, Igh-1a and Igh were persistently up-regulated in the recovery groups. Gene expression of Trem2 also increased 3.44 fold at day 3 compared to day 1 post infection in our model, but we did not detect an increase of immunoglobulins. It is probable that hyperoxia, stainless-steel welding fume exposure and MRSA – mediated pneumonia induce differential lung injuries and signaling pathways.

There are several limitations to our study. First, the mouse model of MRSA – mediated pneumonia does not perfectly mimic pneumonia patients. In the sublethal mouse model of MRSA-mediated pneumonia we used in this study, the mice were able to clear infection within 24 to 36 h, and the infected lungs started to recover [7]. However, the transcriptional events and pulmonary pathological changes during the recovery from infection should be similar in humans and mice. The sublethal mouse model of MRSA – mediated pneumonia could be a good model to study molecular events and mechanisms of host defense and recovery in pneumonia. Second, we examined the global host lung gene expression patterns only at days 1 and 3 post MRSA lung infection. It has been reported that in rodents the expression of cytokines and chemokines was greatest at 6 h after inoculation of MRSA and decreased thereafter [5], [6]. According to our microarray data, expression patterns of many chemokines/cytokines and their receptors such as ccl3, ccl4, cxcl2, il1r2 at day 1 post infection were similar to the ones at 6 h post infection observed in rats [5]. Therefore, lung gene expression patterns at day 1 post infection should be close to the ones at 6 h post infection. Third, this study focused on lung transcriptional events during the recovery and did not examine posttranscriptional and translational modifications in this process. Therefore, inclusion of host lung gene expression patterns at 6 h post infection and examination of posttranscriptional and translational modifications warrant our further study to fully understand molecular mechanisms of recovery after pneumonia.

In summary**,** here we demonstrated considerable gene expression changes in the global gene expression profile occur during the recovery after MRSA lung infection. Eighty two significantly regulated genes were identified, which contribute to recovery from MRSA lung infection by enhancing cell division, cell proliferation, vascularization, and wound healing and adjusting host immune responses. Our data help to understand the molecular mechanisms of how the lung recovers after MRSA lung infection.

## Supporting Information

Table S1 **All genes showing significantly different expression in lungs at Day 3 after MRSA lung infections as compared to the ones at Day 1 after MRSA lung infections.**
(XLSX)
